# Diagnostic and therapeutic challenge of neuroendocrine endometrial carcinoma: a case report

**DOI:** 10.11604/pamj.2024.48.92.36130

**Published:** 2024-07-09

**Authors:** Hariyono Winarto, David Calvin, Fitriyadi Kusuma, Kartiwa Hadi Nuryanto, Yuri Feharsal, Dewita Nilasari, Hartono Tjahjadi

**Affiliations:** 1 Department of Obstetrics and Gynecology, Division of Gynecologic Oncology, Faculty of Medicine, Universitas Indonesia, Dr. Cipto Mangunkusumo Hospital, Salemba, Jakarta, Indonesia,; 2 Department of Health, Faculty of Medicine, Universitas Indonesia, Jakarta, Indonesia,; 3 Department of Obstetrics and Gynecology, Faculty of Medicine, Universitas Indonesia, Dr. Cipto Mangunkusumo Hospital, Salemba, Jakarta, Indonesia,; 4 Department of Anatomical Pathology, Faculty of Medicine, Universitas Indonesia, Dr. Cipto Mangunkusumo Hospital, Salemba, Jakarta, Indonesia

**Keywords:** Neuroendocrine tumor, endometrial carcinoma, radiation therapy, chemotherapy, case report

## Abstract

This article reports a 33-year-old woman with neuroendocrine carcinoma of the endometrium (NECE) with a chief complaint of profuse vaginal bleeding. The patient received emergency radiotherapy to control the bleeding and was discharged. She did not return for four months to undergo a scheduled surgery because she had been hospitalized in another hospital with a COVID-19 infection. She eventually returned due to shortness of breath caused by lung metastasis identified from a chest X-ray. She underwent a total hysterectomy, bilateral salpingo-oophorectomy, and concurrent pelvic and paraaortic lymphadenectomy. The final pathology revealed stage IVB high-grade NECE. The patient died four weeks after surgery from the worsening lung metastases. The aggressive spread, challenging diagnostic nature, and rarity of NECE contribute to the high prevalence of metastasis at the time of diagnosis and poor prognosis. A prospective clinical trial must be performed to formulate an urgently needed guideline for treating NECE.

## Introduction

Endometrial cancer is one of the most common gynecological cancers worldwide [1]. Neuroendocrine tumor is one of its rare variants, comprising only 0.8% of all endometrial cancer cases [2]. Neuroendocrine tumors most commonly occur in the gastrointestinal tract, lungs, and pancreas; they are less commonly found in the gynecological tract. Neuroendocrine carcinoma of the endometrium is highly aggressive; spreading outside the endometrium early in the disease and is thus often detected when it is in its later stages. Additionally, there are currently no standardized treatment recommendations; current treatment relies on recommendations for small cell carcinoma of the lung [3]. There are approximately only 150 cases of NECE reported in the literature [4]. The rarity of the disease poses a diagnostic and therapeutic challenge. This report discusses the case of a 33-year-old diagnosed with NECE with rapid progression.

## Patient and observation

**Patient information:** our patient was 33 years old, parity 0. She had a year-long history of frequent irregular vaginal bleeding, and she changed her pads about four times a day during the year. She also complained of severe weight loss and appetite loss. She had type II diabetes mellitus diagnosed one year ago, controlled by insulin and antidiabetics. She also had hypertension diagnosed six years ago. There was no familial history of cancer.

**Clinical findings:** the patient arrived with profuse bleeding. Her general condition and vital signs were stable, with a slight increase in heart rate of 104 per minute. However, her gynecological examination revealed continuous vaginal bleeding and a mass protruding from the cervix with several necrotic parts.

**Timeline of current episode:** the case presentation organized as a timeline can be seen in Table 1.

**Table 1:** case presentation organized in a timeline

Date	
October 2020	**First hospital:**
	The patient complained of irregular vaginal bleeding.
	Diagnosed with abnormal uterine bleeding in the first hospital.
	Treated with transfusions and several drugs that did not relieve the symptoms.
December 2020	**Second hospital:**
	**Gynecologic examination:** protruding mass from the cervix
	**Histopathologic analysis:** poorly differentiated adenosquamous carcinoma of unknown origin.
	Referred to our gynecology oncology clinic.
March-April 2021	The patient arrived at our clinic with profuse bleeding
	**Gynecologic examination:** continuous vaginal bleeding and protruding mass from the cervix with several necrotic parts.
	**Ultrasound examination:** tumor mass filling the vagina with increased blood flow
	The patient was treated with emergency radiation applied in five fractions with single doses of 4 Gray to control the bleeding.
**May 2021**	Post-radiation ultrasound examination: the mass was infiltrating over half of the myometrium originating from the endometrium.
	**Diagnosis:** stage IB endometrial cancer.
	The patient was planned for a hysterectomy.
**The patient could not be contacted for three months**	
August 2021	The patient arrived at the emergency room due to unbearable abdominal pain and shortness of breath.
	**Chest X-ray:** metastasis to both lungs was found (multiple nodular opacities in both lungs)
	**Abdominal MRI:** metastasis to the para-iliac lymph nodes was found.
	**Diagnosis:** stage IVB endometrial cancer.
12^th^ October 2021	A hysterectomy was performed with bilateral salphyngoophorectomy, pelvic lymphadenectomy, and paraaortic lymphadenectomy.
	**Histopathologic analysis:** high-grade neuroendocrine carcinoma with poor differentiation and positive lymphovascular space invasion
	Immunohistochemistry staining: positive for synaptophysin, chromogranin, and CD56.
31^st^ October 2021	The patient complained of severe coughing for one week.
	The patient was planned for chemotherapy and radiotherapy regimens.
November 2021	The patient experienced severe shortness of breath and died before being transported to our emergency center.

**Figure 1:**
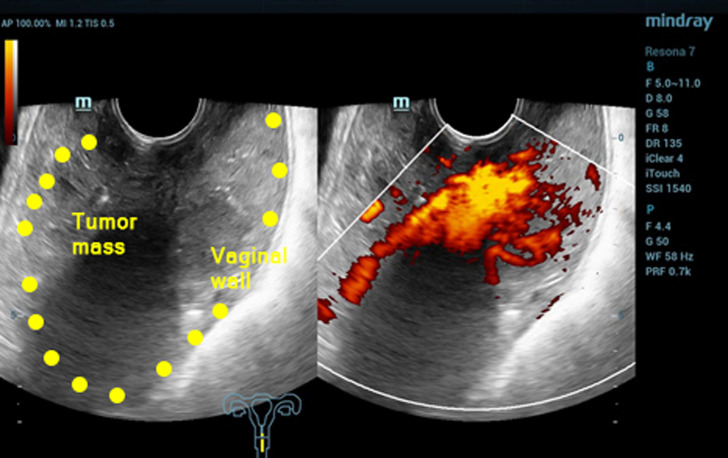
first ultrasound imaging before radiotherapy; the yellow dots indicate the tumor mass' borders

**Figure 2:**
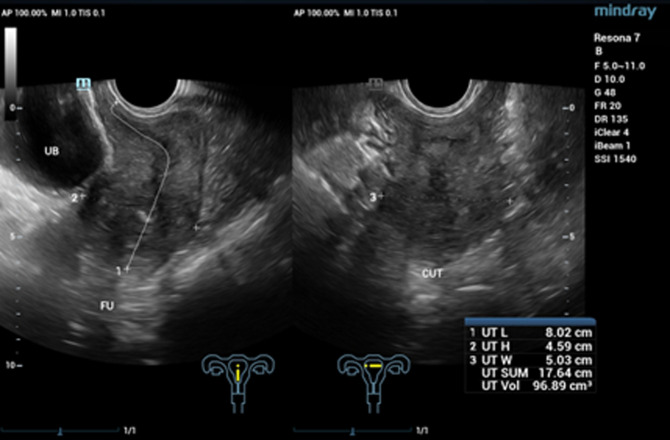
second ultrasound imaging of the patient after radiotherapy

**Figure 3:**
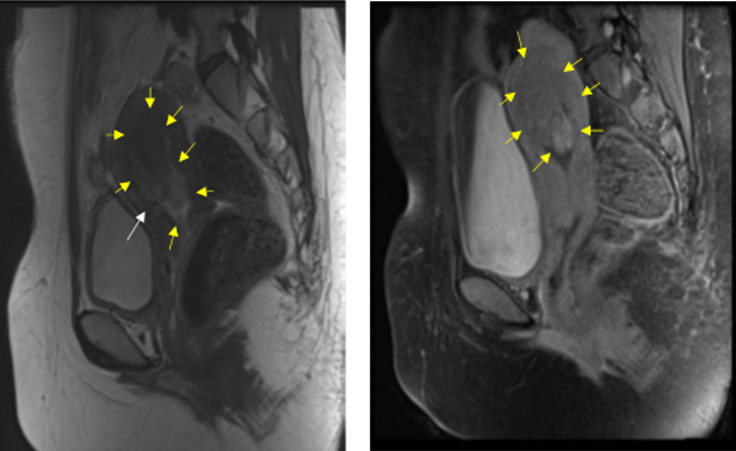
MRI after radiotherapy; the yellow arrows indicate the tumor which has reduced in size, no longer protruding through the cervix

**Diagnostic assessment:** six months prior, she started to seek medical help at a hospital due to several episodes of heavy bleeding. Her hemoglobin level was only 6 mg/dL. She had received transfusions and several drugs but failed to relieve the symptoms. She was then diagnosed with abnormal uterine bleeding. Two months of treatment at the first hospital did not relieve her symptoms. She then came to another hospital. On physical examination, a mass protruded from her cervix, with histopathologic results revealing poorly differentiated adenosquamous carcinoma with unknown origin. Recognizing the diagnosis, she was referred to our gynecology oncology clinic. Ultrasound examination showed a tumor mass filling the vagina with increased blood flow (Figure 1). The laboratory result showed microcytic hypochromic anemia with a hemoglobin level of 8.4 mg/dL and hypoalbuminemia with an albumin level of 2.96 mg/dL. The tumor was suspected to be malignant. She was then treated with emergency radiation therapy applied in five fractions with single doses of four Gray to control her bleeding. Following her regimen of emergency radiation, she was re-examined with an ultrasound examination. It was found that there was a mass infiltrating over half of the myometrium originating from the endometrium. She was then diagnosed with stage IB endometrial cancer (Figure 2). She was also examined with magnetic resonance imaging (MRI). Her MRI result was a solid endometrial mass infiltrating the anterior myometrium at the uterine fundus (Figure 3). Following the radiotherapy, her tumor decreased in size, and bleeding ceased.

**Figure 4:**
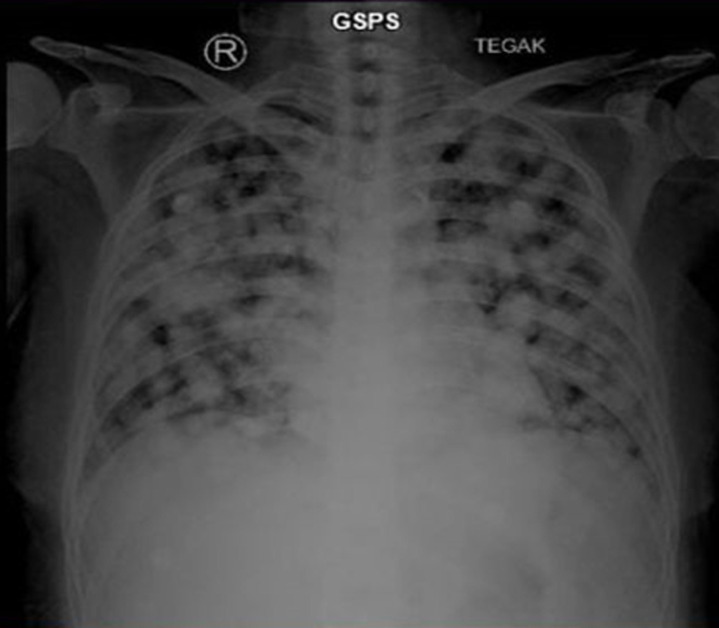
chest X-ray revealing metastasis to the lungs

**Diagnosis:** stage 1B endometrial cancer. A hysterectomy was planned immediately. Soon after, she contracted COVID-19 and had to be hospitalized for two weeks. After her COVID-19 infection resolved, she further delayed her treatment due to fear of contracting COVID-19 again. She could not be contacted for three months following her hospitalization. Nevertheless, she finally came to our emergency room due to unbearable abdominal pain and shortness of breath. A chest X-ray and whole abdominal MRI revealed that cancer had spread into the lungs and para-iliac lymph nodes, changing her diagnosis into stage IVB endometrial cancer (Figure 4).

**Figure 5:**
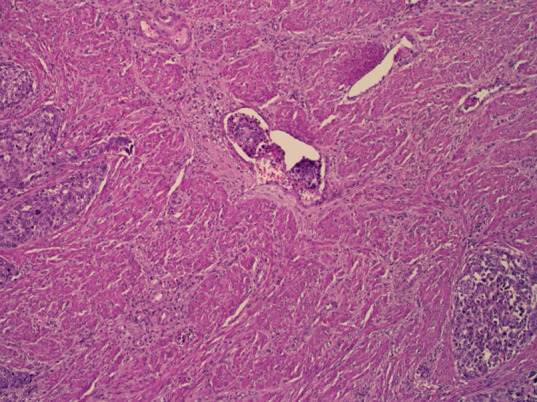
lymphovascular space invasion

**Figure 6:**
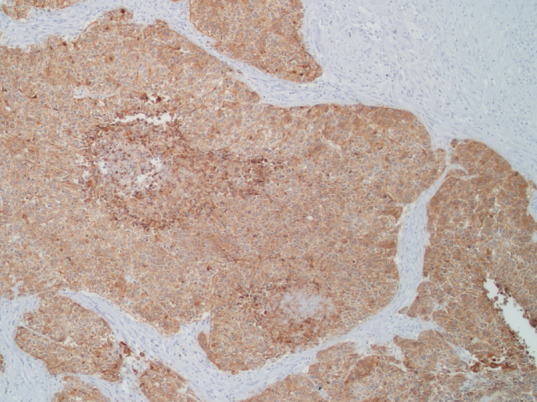
positive stain for synaptophysin

**Figure 7:**
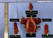
specimens of the uterus, adnexa, bilateral pelvic lymph nodes, and paraaortic lymph nodes

**Therapeutic interventions:** afterward, a hysterectomy was performed with bilateral salpingo-oophorectomy, pelvic lymphadenectomy, and paraaortic lymphadenectomy. Histopathologic results revealed high-grade neuroendocrine carcinoma with poor differentiation and positive lymphovascular space invasion (Figure 5). Infiltration and metastases to the left fallopian tube and right pelvic lymph node were observed. Immunohistochemistry staining was positive for synaptophysin (Figure 6, Figure 7), chromogranin, and CD56, confirming the diagnosis of neuroendocrine endometrial carcinoma.

**Follow-up and outcome of interventions:** on the third day following the surgery, she suffered a surgical site infection, treated with re-stitching. She was discharged the day after. Two weeks following her discharge, the patient complained of severe coughing for one week. She was planned for chemotherapy and radiotherapy sequential regimens. However, a month following her last visit, she had severe shortness of breath. Unfortunately, she died before being transported to our emergency center.

**Informed consent:** written informed consent was obtained from the family to publish this case report and accompanying images.

## Discussion

NECE comprises of only 0.8% of endometrial cancer cases. The rarity of neuroendocrine tumors presents a diagnostic challenge, where an accurate diagnosis is crucial for determining a treatment plan and prognosis. Metastasis is commonly found at the time of diagnosis, with 56-68% percent of patients presenting with The International Federation of Gynecology and Obstetrics (FIGO) stage III or IV disease. This type of tumor is likely to be highly proliferative and has spread outside the endometrium at an early stage [3].

Histopathologically, neuroendocrine endometrial carcinoma exhibits the prototypical morphological features of small cell carcinoma: highly atypical cell sheets with scant to imperceptible cytoplasm, egg-like to slightly spindle-shaped nuclei hyperchromatic and scattered chromatin, and inconspicuous nucleoli. Moreover, nuclear molding, multiple mitoses, apoptosis, extensive necrosis, and crush artifacts are frequently seen. Therefore, immunohistochemical staining with chromogranin, synaptophysin, and CD56 are proposed to be the diagnostic criteria for neuroendocrine endometrial carcinoma as they can establish the tumor's neuroendocrinological nature [5]. A retrospective study of 25 NECE cases revealed that 89% disagreed with its original diagnosis; misinterpreted as undifferentiated carcinoma, a sarcomatoid component of carcinosarcoma, endometrioid carcinoma, primitive neuroectodermal tumor, and carcinoid tumor [6]. This can be reflected in our case, histopathological results were initially interpreted as adenosquamous carcinoma and finally diagnosed as NECE after surgery.

There are currently no guidelines for treatment strategies for endometrial neuroendocrine carcinoma. What is now available in the literature are case reports and case series. Several studies have suggested treatment using surgical resection, radiotherapy, and platinum-based chemotherapy (cisplatin), all based on small cell lung cancer treatment. No extensive studies or prospective clinical trials have been conducted to guide the therapy in neuroendocrine endometrial carcinoma. For this histopathologic type from tumors on other organs, the proposed drug is etoposide and platinum-based chemotherapy [3,6]. A case series of 25 patients by Pocrnich *et al*. reported long-term survival of patients with FIGO stage III. Out of the ten cases with FIGO stage III, six cases achieved long-term survival with a median of 79 months without evidence of disease. Three of the five patients with FIGO stage I disease attained more than 60 months without evidence of disease, while one achieved 37 months without evidence of disease. The remaining 12 patients died of disease, while survival data of the remaining three patients were unavailable. All the patients without evidence of disease received a combination of hysterectomy, platinum-based chemotherapy, and radiotherapy, except for two patients who received only a hysterectomy (FIGO stage IA) and a hysterectomy and radiotherapy (FIGO stage IB) [6] Schlechtweg *et al*. who retrospectively studied 364 women with NECE revealed that radiotherapy showed no reduction in the risk of death. Chemotherapy, however, has significantly reduced the risk of death. In our case, radiotherapy was done to control the profuse bleeding symptom [3]. However, a prospective clinical trial is required to study the efficacy of radiotherapy in treating NECE.

Studies have reported the use of targeted therapy for other types of neuroendocrine tumors. One example is the use of phosphoinositide 3-kinase (PI3K) inhibitor, cisplatin, and etoposide combination, which significantly reduced cell growth of cervical neuroendocrine carcinoma [7]. The use of programmed cell death protein 1 (PD-1) inhibitor has also been combined with nivolumab to treat FIGO stage IB2 cervical neuroendocrine carcinoma, showing complete resolution after six doses of 3 mg/kg IV every two weeks [8]. Unlike neuroendocrine tumors of the gastrointestinal tract and lungs, the rarity of NECE itself may become an obstacle in having a prospective clinical trial performed [3].

Despite our patient being first diagnosed with FIGO stage IB endometrial neuroendocrine carcinoma, poor compliance due to loss of follow-up has hindered timely treatment of the disease, allowing further spread of the disease and worsening an already poor prognosis of NECE. The fear of contracting COVID-19 in healthcare facilities during the height of the pandemic has been studied. Montalto *et al*. reported that more than half of surgery patients are concerned about contracting COVID-19 at the hospital [9]. He and colleagues reported a significant association between delayed breast cancer treatment with fear/anxiety levels concerning COVID-19, which includes chemotherapy, radiotherapy, targeted therapies, and endocrine therapy [10].

## Conclusion

NECE is a rare form of endometrial carcinoma. Due to its rarity, the aggressive spread of disease, and challenging diagnostic nature, it is prevalent for metastasis to have already occurred at the time of diagnosis in patients with neuroendocrine endometrial carcinoma. There are no guidelines for treating NECE; current treatment approaches are based on data for neuroendocrine carcinoma of the lung. A prospective clinical trial must be performed to formulate an urgently needed guideline for treating NECE.
